# Thoroughness

**Published:** 1885-10

**Authors:** L. P. Dotterer


ARTICLE V.

### THOROUGHNESS.

BY L. P. DOTTERER, D. D. S.

[Read before the South Carolina Dental Association.]

Though scarcely more than a novice in the vast field of Operative Dentistry, I have gleaned sufficient experience from observation and practice to know that THOROUGHNESS is the surest means of success.

Just as the tillers of the soil sow their seeds, watch their crops, and reap their harvests, so must we do our duty, advise our patients as to the best means of preservation, and would that I could say, reap our harvest. There has been so much written upon this subject that I have nothing *new* to say, but will touch upon several points, and in giving my idea of thoroughness, as there applied, I may draw out some discussion.

The first step towards the preparation of the mouth for dental operations is the removal of calculus and decayed fangs. Let this be done in a manner that will *insure future cleanliness*, where the proper after attention is given on the part of the patient.

As regards the preparation and filling of cavities, there are so many conflicting conditions, that we must be governed entirely by the case before us; but to be thorough in our preparation, we must so shape the cavity as to have the walls nearly plumb, uniform margin, slightly undercut. In proximal cavities there may be a groove or pit at cervical wall, but do not have it too near the margin, on account of its liability to produce fracture, and consequent failure at that point. On grinding surfaces, cut out all fissures leading into cavity, and be careful to have no angles.

The margin, after all, is the most important point; for just here failure begins, especially at the cervical wall, and care should be taken to thoroughly remove all softened structure, and aim to reach a solid foundation. These margins should be carefully trimmed and burnished, and thus our cavity is ready for the filling.

We often hear practitioners decry the rubber dam, and boast of their skillful use of the napkin; but, gentlemen, many are the failures consequent! For in deep proximal cavities, the dam is invaluable in keeping guard against oozing moisture from the gums, which, without this precaution, will flow upon the filling without our knowledge.

The dam adjusted, we proceed to form a mass of non-cohesive gold, and where the walls are strong enough, we can continue with this material throughout. But where cohesive gold is necessary, we should cover our borders, as far as possible, with soft foil; for this is more adaptable to the walls. Another advantage to be found in non-cohesive gold, is its pliability, ease of starting, and rapidity in finishing. We should thoroughly condense from beginning to end, whatever may be the kind of foil used.

Filing and finishing is too often hurried through, leaving a surplus of material at the cervical wall, or lapping the edges—another sure cause of failure; and every care should be directed to finish in such way that an instrument passing over the line of demarkation cannot detect it. After filing, we would use pumice, either on a strip of orange-wood, or by some other convenient means, and then polish. The same general rule holds good in amalgam work, and the main cause of failure in these cases is that lack of thoroughness in finishing.

In grinding-surface cavities, where the enamel leading thereto is funnel-shaped, we often introduce too much amalgam, extending it beyond the margins of the cavity, and finishing to a fine edge. This material, when hard and bit upon, will fracture perpendicularly around the margins, giving the finishing a bulged appearance, and exposing a V-shaped crack, which will invite decay. Consequently, we should remove all surplus material, and finish at the very margin of the cavity. When gold is used, this precaution is not so necessary, as the edges of a gold filling will not fracture. Since we do not have to mallet amalgam, it is natural to suppose we don't require firm margins, but this is a mistake; and as much, or even more care should be exercised in the preparation of a cavity for amalgam than gold, as tooth-structure seems to waste away more rapidly from the former.

Let our motto be, "Whatever is worth doing at all is worth doing well." If applying arsenic or a disinfectant, cover it with gutta-percha, for the patient may be delayed a few days longer than we anticipate; and what is worse than removing a foul piece of cotton, and finding the tooth in a poorer condition than we left it? If we introduce a temporary stopping on account of exposure or frailty, let it be done thoroughly; and after relating its importance to the patient, caution her to return at a certain time for its removal and permanent filling.

We must be teachers at our chairs, if we wish the public to appreciate us, and we should instruct patients in the proper care of their teeth by an intelligent and thorough use of the brush, pick, etc.

Such is the importance of thoroughness in dental operations. This paper does not half express it, but for fear of trepassing too much on your valuable time, I commend these ideas to your criticism.—*Southern Dental Journal.*

